# Supplementary material for: Shapify: Paths to SARS-CoV-2 frameshifting pseudoknot
Source: PLoS Comput Biol. 2023 Feb 28;19(2):e1010922. doi: 10.1371/journal.pcbi.1010922 (PMC10004594; doi:10.1371/journal.pcbi.1010922)
Supplement: Attachment — Submitted filename: Response_to_Reviewers_Oct_04.pdf [file pcbi.1010922.s002.pdf]

Response to Reviewers

October 4, 2022

We would like to thank the reviewers for their time and constructive feedback. We have revised the manuscript based on the feedback. Major revisions: (1) Inclusion of an additional *in vitro* SHAPE dataset to obtain secondary structure predictions; (2) Analysis of MERS-CoV mutated sequence structure predictions (3) Comparison between our proposed algorithm Shapify and existing method ShapeKnots. In the revised manuscript, all additions have been highlighted in blue. We adjusted the abstract and author summary. The introduction and background sections were combined. Methods, results, and discussion were restructured for clarity. Extraneous information was omitted and responses to reviewer comments were added. Following is our point-by-point response to the comments, including a description of any additional experiments that were carried out, in blue.

Reviewer 1

In this manuscript, the authors develop an algorithm Shapify to predict RNA secondary structures with SHAPE reactivity data constraints, following the RNA hierarchical folding hypothesis. Namely, energetically favorable initial stems predicted by HotKnots, together with modified thermodynamic parameters by SHAPE data, are used to guide pseudoknotted predictions by HFold. The authors then apply Shapify on SARS-CoV-2 frameshifting element to identify the native 3-stem H-type pseudoknot and many non-native structures. They further derive pathways to relate the initial stems and the predicted 2D structures. Moreover, they study the impact of natural FSE mutations on the structural stability, predict a consensus structure for SARS-CoV-2, SARS-CoV, and MERS-CoV FSEs, as well as 2D structures for MERS-CoV FSE.

Despite the many aspects covered in the manuscript, the different sections need to be pieced together to deliver meaningful implications. The Methods section lacks clarity, and the Results section needs much more in-depth analysis. The necessity of Shapify is not convincing enough, as ShapeKnots can also achieve similar tasks. Comparisons with other studies that present alternative FSE structures are needed. Overall, the manuscript requires major revisions to be considered for publication.

Response: We thank the reviewer for the detailed feedback. Following we respond to each comment separately.

Below are the detailed comments:

1. In Introduction, page 3, the authors mention the hierarchical folding hypothesis. Instead of saying "the hierarchical folding hypothesis is supported by experts [17-19]", can the authors briefly state how it is supported in these three studies.

   Response: We have significantly revised our manuscript and moved the definition of hierarchical folding hypothesis to a more suitable place, Materials and methods, Background on RNA Structure Prediction: "This hypothesis posits that RNA first folds into a pseudoknot-free structure, that is, one without crossing base pairs. Then, additional bases pair that may form pseudoknots to lower the minimum free energy of the structure [50]. The initial structure may be modified locally to accommodate formation of more stable base pairs [51, 52]. Hierarchical folding paths were experimentally identified in multiple pseudoknots [53], including frameshifting pseudoknots [54]. "

2. In Introduction, page 3, the authors claim that "Potential non-native secondary structures for the SARS-CoV-2 -1 PRF pseudoknot have not been accounted for in recent analyses [23,24]". However, many recent SARS-CoV-2 FSE papers have proposed alternative 2D structures, such as Huston et at., Schlick et al., Manfredonia et al., etc. The authors should compare these alternative structures to the non-native structures predicted here.

   Response: In the revised manuscript we added the following text to the Introduction to provide a more comprehensive background on the recent efforts for determining alternative structure: "The structural model proposed for the SARS-CoV-2 -1 PRF pseudoknot continues to evolve. Huston et al. [12] found complex folding dynamics with multiple conformational clusters. In [13, 14, 15], additional structures for the pseudoknot have been proposed. Furthermore, Yan et al. [16] suggest generalized structure motifs that may be affected by unfolding dynamics during translocation. However, there has not yet been a focus on identifying the ensemble of energetically favourable structures in proximity to the minimum free energy structure of the pseudoknot. Given that non-native folding paths correlate with -1 PRF efficiency and are relevant to structure function, further research on identifying energetically favourable SARS-CoV-2 -1 PRF pseudoknot conformations is valuable in structural prediction."

3. In Introduction, page 4, the authors claim that "To the best of our knowledge there is currently no computational method available for prediction of complex suboptimal pseudoknotted secondary structures with SHAPE data". However, ShapeKnots can perform this task by modifying its option of maximum number of structures predicted, or option of maximum percent differences in folding free energy change for generating suboptimal structures. Moreover, ShapeKnots also allows the users to specify initial constraints on nucleotide base pairing. Please double check and compare the Shapify prediction results with ShapeKnots to show why we need Shapify.

   Response: We note that ShapeKnots is a heuristic method, so it does not guarantee finding the minimum free energy structure or in case of suboptimal structures, structures that are energetically close enough to the minimum free energy structure. In our revised manuscript we dedicated a section to comprehensively compare performance of ShapeKnots with our proposed method, Shapify, both to have a baseline for our comparison and to justify the need for Shapify. We ran ShapeKnots with and without our identified initial stems (the same we used for Shapify), and as expected found ShapeKnots unable to identify as many low free energy structure as Shapify is able to predict. In addition, ShapeKnots predictions were not found to effectively utilize the initial stems (i.e., ShapeKnots performance was better without the initial stems).

4. In Methods, subsection "Secondary Structure Prediction", can the authors justify why they choose HotKnots to do the initial stem predictions? For HFold, does it only allow one initial stem as input constraint? If not, how about incorporating any pseudoknot-free initial structure as constraint for Shapify, instead of allowing only one initial stem?

   Response: To predict the structure of SARS-CoV-2 and MERS-CoV in the absence of SHAPE data, we used Iterative HFold. Iterative HFold takes as input an RNA sequence and a pseudoknot-free structure to predict a possibly pseudoknotted secondary structure guided by the input structure through the hierarchical folding hypothesis. As noted by Jabbari and Condon [56], the method performs well using low free energy (stable) pseudoknot-free stems as input structure. Here we used multiple runs of Iterative HFold (in case of no SHAPE information) and Shapify (with SHAPE reactivity information) each time using one stable initial stem to sample the structural pathways to final prediction. We note that since Iterative HFold is capable of modifying original input structure to accommodate lower free energy base pairings, multiple input structures can result in the same predicted output structure. This is the same for Shapify. This experiment allows us to assess accessibility of native and native-adjacent structures from different starting points.

5. In Methods, subsection "Shapify Method", what exactly are the "nearest-neighbor model parameters [64]"? Are SHAPE reactivity data incorporated during the HFold prediction step? The citation [64]

there is for HotKnots, very confusing. Please clearly state the steps in Shapify using a flowchart like Fig. S9, with SHAPE constraints included.

Response: In the revised manuscript we clarified explanation of our Shapify method. In particular we explained that: "Shapify takes as input an RNA sequence, a SHAPE dataset, and a pseudoknot-free secondary structure (cf. supplemental file 1, Fig. S11: Shapify Hierarchical Folding Pipeline) and outputs the predicted secondary structure. Both SHAPE data and the pseudoknot-free input structure guide Shapify's prediction with known RNA structural information. We used the pseudo energy terms created by Deigan et al. [62] from SHAPE reactivity data, as a means of integrating such data into our prediction algorithm."

6. In Methods, subsection "SHAPE data", please mention that all three available SHAPE datasets are genome-wide probing. Table 1 and Figure 6 are presented here, which should go to the Results section. Please reorganize this subsection to incorporate it better with the Methods and Results sections.

   Response: We have re-organized the section based on your suggestion. We further included the data in Table 2 of the revised manuscript to improve readability and accessibility.

7. In Results, subsection "Betacoronavirus -1 PRF Pseudoknot Structural Similarity", the authors mention "RNA-class probability" calculated by RNAz. Please explain what exactly this probability is. In Table 2, besides the consensus 2D structure prediction by RNAz, please also show the multiple sequence alignment by Clustal.

   Response: In the Results subsection "-1 PRF Structural Similarity" of the revised manuscript, we removed the confusing term and instead added its meaning and implication. In addition we added the consensus sequence and alignment to the new Table 3, as per the reviewer's suggestion.

8. In Table 3, the authors should list other popular stems mentioned in subsequent text. Checking the Supplementary Information every time these stems mentioned is exhausting.

   Response: We thank the reviewer for their suggestion. All important stems are named and are included in the main text table.

9. In Results, subsection "SARS-CoV-2 -1 PRF Pseudoknot", the authors argue that different initial stems can lead to the same structure prediction. In particular, using stems 1, 3, 9, 18 all give the native structure. However, stems 1 and 18 are not compatible, and stem 18 is not included in the native structure. Similar for stems 3 and 9. Hence, it seems that the initial stems are not completely included in the final predictions, just as guidance? Then to what extent are these initial stem constraints used in the predictions? The authors need to clarify and justify this.

   Response: As we noted above, following the flexible hierarchical folding hypothesis as implemented by Iterative HFold and Shapify, original base pairings may open up to accommodate formation of lower energy base pairs. This is the reason, why some of the initial stems are not part of the final structure while they lead to the formation of the final structure. We have included this point in Materials and Methods, under Background on RNA Structure Prediction.

10. In Results, subsection "SARS-CoV-2 -1 PRF Pseudoknot", the authors only describe the native FSE structure. Please also analyze the other structures predicted and shown in Table 4.

    Response: We have done extensive restructuring and revision clarifying our contributions and discussing our results better as per the reviewer's suggestion. Current Subsection "SARS-CoV-2 -1 PRF Pseudoknot" under Results presents non-native structures, as well as the native structure. We have gone further in our classification and discuss two types of non-native structures, one we call native-adjacent, which refer to structures that are close to the native structure, and the second we call native non-adjacent, meaning structures that are markedly different from the native structure.

11. In Results, subsection "SARS-CoV-2 -1 PRF Pseudoknot", for the mutated sequence predictions, the authors can show where these mutations locate in the native structure, and list their impacts on the frameshifting efficiencies, along with Table 5. The stem labeling is very confusing, what are a, b, c? Suboptimal stems are mentioned a lot here, but they are only presented in the Supplementary Information without any descriptions. This makes this subsection hard to comprehend.

    Response Thank you for your feedback. To clarify the section, in our revised manuscript we moved mutation results to "Effect of Mutations on the SARS-CoV-2 -1 PRF Pseudoknot" subsection. We have added red arrows to label the location of mutations in Fig. 2. To clarify the use of letters (a, b, c), we further added the explanation to the section: "Initial stems for mutated sequences are given an ID with a letter to distinguish them from the reference sequence stems (e.g., $5a$). If multiple stems from mutated sequences have the same free energy ranking with respect to the initial stems of the reference sequence, they are given IDs with sequential letters (e.g., $5a$ and $5b$)."

12. In Results, subsection "SARS-CoV-2 -1 PRF Pseudoknot with SHAPE", the authors say that "In some cases, more than three predictions were obtained for a single initial stem, due to suboptimal structure prediction". When would suboptimal predictions be included, using certain free energy threshold? Also, "Darker color represents higher agreement between SHAPE data", how is this agreement measured?

    Response: In the revised section we explained: "There are four predictions obtained for each initial stem, one for each of the SHAPE datasets used. Note that predictions among the four results could be the same. Here we include any additional suboptimal structures within 2 kcal/mol of the minimum free energy prediction. Darker color represents higher agreement among SHAPE datasets, meaning the same prediction was obtained with different SHAPE data." We further explained the colour darkness in the caption of Fig. 6 and 7: "If the structure predicted for a specific initial stem was the same for all four SHAPE datasets it is presented with a black colored path. In other cases, where the predicted structure was the same for three, two, or only one of the SHAPE datasets, the path is colored dark grey, grey, or light grey, respectively."

13. In Results, subsection "SARS-CoV-2 -1 PRF Pseudoknot with SHAPE", Figures 7 and 8 need more analysis and interpretation. In the figures, why are there many blank spaces among the predicted structures? Also, it would be helpful to show the initial stems alongside.

    Response: We have revised the figures to include an *in vitro* SHAPE dataset to improve over discussion on structural similarity. We have included initial SARS-CoV-2 -1 PRF stems in the main text of the revised manuscript, but due to width-limitation of the figures, we were not able to fit the stems as part of the figures as suggested by the reviewer. We focus more on structural paths in the Results and Discussion sections of the revised manuscript.

14. In Results, subsection "MERS-CoV -1 PRF Pseudoknot", why not compare the initial stem and 2D structure predictions with those of SARS-CoV-2?

    Response: Thank you for the great comment. We have included comparison with SARS-CoV-2 initial stems to the revised manuscript.

15. In Results, subsection "SARS-CoV-2 -1 PRF Pseudoknot SHAPE Data Analysis", please reorganize this subsection with the Methods "SHAPE data". Please indicate the nucleotides that have significant differences in ShapeKnots and Shapify bootstrap tests in Figure 6.

    Response: As per the reviewer's suggestion we have reorganized the section and compared the predictions of Shapify and ShapeKnots explicitly.

16. In Discussion, page 18, when mentioning the Schlick et al. studies, note that 3_6 is a coarse-grained representation of the native 3-stem H-type pseudoknot, invariant to the stem lengths. NN1, 2, 4 structures all correspond to 3_6. Please revisit their studies.

Response: Thank you for your comment. We have revised our manuscript to state the contribution of Schlick et al. more clearly.

17. In Discussion, page 18, the statement "while ShapeKnots is solely reliant on SHAPE data to guide prediction, Shapify can better capture the conformational flexibility of RNA because it is also guided by partial structure information" is not completely true. ShapeKnots can also take initial base pair constraints. Please double check.

    Response: In the revised manuscript we dedicated one section to better explain the results obtained using ShapeKnots. As mentioned in this section, we ran ShapeKnots once with only SHAPE data and once again with SHAPE and the same initial stems that we used for Shapify. ShapeKnots's predictions were better when initial stems were not used. We have further compared ShapeKnots' predictions with Shapify's and observed that in general structures predicted by Shapify have lower free energy compared to those predicted by ShapeKnots.

18. In Discussion, page 19, is the statement "SHAPE data may reflect secondary structure that forms between the sequence of interest and upstream bases; however, such structures would not be relevant to frameshifting, which occurs when the ribosome is in place over the slippery sequence" absolutely true? Stems formed with upstream bases can be involved in structure transitions during ribosomal unwinding, which could in turn impact the ribosomal translation and frameshifting. The authors can check other studies that focus on alternative FSE structures, such as Schlick et al. and Manfredonia et al.

    Response: Thank you for your comment. In the revised manuscript, we clarified the point and added an *in vitro* SHAPE dataset to account for possible structures that span across our targeted region.

19. In Discussion, page 19, in the citation of Zhang et al. work, "Of interest is that Zhang et al. identified the same 3D RNA structure in a range of betacoronaviruses including MERS-CoV, SARS-CoV, and SARS-CoV-2", need to clarify that these are computational models, not Cryo-EM. Also, correct the 3_6 reference as mentioned above.

    Response: We have revised our Discussion section accordingly.

20. A recent study by Neupane et al. (Nat. Commun., 2021) uses optical tweezers to show the folding pathway of SARS-CoV-2 FSE. The authors should cite and relate with this study.

    Response: Thank you for the information. We have added the study and its contributions to our revised manuscript.

21. The Figures need higher resolutions.

    Response: Higher resolution figures are included with the revised manuscript.

Reviewer 2

Here Trinity et al. explore the conformational space of frameshift stimulatory pseudoknots from coronaviruses using an algorithm they introduce (called Shapify) that combines base-pairing predictions and SHAPE reactivity data. They predict a range of conformations that differ from the putative native structure, and look in particular at the effects of starting the folding with different, sub-optimal hairpin structures. Intriguingly, they find that the native structure is somewhat robust against starting from sub-optimal hairpins, with multiple paths leading back to the "correct" expected structure, but non-native structures generally form via unique paths. The analyses are applied primarily to the pseudoknot form SARS-CoV-2, with subsets also applied to the pseudoknots from MERS-CoV and SARS-CoV.

The problem of pseudoknot structure prediction is an important one, and prediction tools remain somewhat unreliable. Hence a new prediction algorithm that has been validated to work better than existing tools would be a valuable addition to the literature. The work presented here looks promising, but it's unclear how much of an improvement it represents over currently available methods, because the authors have not provided a comparative analysis of its performance. Revisions are therefore needed before suitability for publication in PLoS Computational Biology can be assessed meaningfully.

Response: We thank the reviewer for the detailed feedback. Following we respond to each comment separately.

Issues for the authors to address in revision:

1. If a new approach is being proposed in a field where other methods exist, one would generally expect to see some evaluation of the performance of the new methods in comparison to previous methods: does it give more reliable results, require fewer computational resources, or have some other advantage over previous methods? There is some qualitative discussion of this issue, but it is not approached in a systematic way.

   Response: Thank you for your feedback. We have included a systematic comparison with the only existing method that is capable of incorporating SHAPE reactivity data and predicting pseudoknotted structures (ShapeKnots), as part of our revised manuscript. This we believe provides a baseline for comparison and justifies the need for our proposed method, Shapify.

2. On a related topic: the authors have structured the main text around the interesting things they learn about coronavirus pseudoknots, but the motivation for the work in the Author Summary is that there is a need for better structure-prediction tools. There seems to be some confusion about what is the main point of the manuscript: is the focus on introducing the Shapify method, or is the focus on the results it produces? If the former, the paper needs to evaluate the comparative performance of the method; if the latter, then the performance may be left out as not in scope. However, given that this manuscript is the first one describing the method, and given the authors' own motivation for the paper, it would seem to be necessary to focus on the method.

   Response: As per the reviewer's comments, we restructured our work to better motivate the main contribution of the paper.

3. Adding to the confusion about the purpose/focus of the paper, the authors presented an exceptionally detailed overview of frameshifting and pseudoknots in the introduction and background sections (which is rather more comprehensive than probably needed!), but strangely they did not provide an overview of the different approaches to structure prediction for pseudoknots and their various pros/cons. The latter would seem to be important for placing the work here in context. There are a number of approaches to be discussed, including methods that incorporate constraints from SHAPE data, reviewed for example in Li et al. (2020) Front Genet 11:574485 (among other reviews).

   Response: Thank you for your comment. We have focused the Introduction better in the revised manuscript. We note that the work of Li et al. focuses on RNA tertiary (3D) structure prediction while the focus of our paper is on secondary structure prediction. In the new "RNA Structure Prediction" subsection of Materials and methods, we explained this point better and cited the work of Li et al.

4. The use of several different SHAPE datasets is a strength of the analysis. It's worth noting, however, that in vivo and in vitro SHAPE analyses don't always agree, and it's not clear which is correct (a number of in vivo analyses have suggested that pseudoknots are not present, for example, which is very unlikely to be correct, and most likely reflects some problems with the SHAPE assay). It would be a good idea to include some in vitro SHAPE datasets. One example is the SHAPE data from ref. 33. An advantage of including this dataset is that it accompanies the cryo-EM structure, which provides

a form a validation for the predictions (although the cryo-EM structure is solved from just a small subset of all the images).

Response: As per the reviewer's suggestion we added an *in vitro* SHAPE dataset to our analysis. We further revised our analysis (see the revised "SARS-CoV-2 −1 PRF Pseudoknot SHAPE Data Analysis" and the Discussion section corresponsing to "Bootstrap") to include the possible disagreements of the datasets.

5. Regarding the question of validating the method, it's not obvious how to proceed. There are a few conceptual problems that can be foreseen: (i) Predictions of sub-optimal structures are hard to validate, because most experimental methods see the dominant structure only. Analysis of minority structures in the cryo-EM imaging might provide an avenue to explore this, but such an analysis does not seem to be available yet. Validation may therefor have to depend solely on the optimal structure. (ii) Differences in the SHAPE data (one of which disagrees very markedly!) raise questions as to how reliable the "constrains" they provide really are! However, the fact that the method seems to come to a consensus result (if I understood it correctly) is reassuring. This issue could be discussed more clearly (how and why it is able to do so). (iii) How should one understand conflicts between the predicted structures and the SHAPE reactivity? One problem is that SHAPE reactivity measures not whether a base is paired, but rather whether the backbone is flexible, hence an unpaired base that is involved in tertiary interactions (not predicted here) might not be reactive. In contrast, bases that are paired should be non-reactive, hence a disagreement here presumably suggest the prediction is wrong.

   Response: We expanded our discussion on SHAPE data and analysis in the revised manuscript to address the points raised by the reviewer better.

6. On a related note: the exploration of alternative/non-native structures is one of the most interesting parts of the work, but is there any evidence experimentally for these structures? Some of them are predicted to have energy differences of only 1–2 kT, so thermodynamic arguments suggest they should be present in modest abundance. If they are not seen, perhaps it's because the methods used experimentally select in some way for particular structures, or maybe there are kinetic reasons? The question of experimental evidence (and how it might be obtained) should be discussed.

   Response: As pointed out by the reviewer, proving existence of suboptimal structures is not an easy task. Here by exploring the free energy landscape, we argued for possible paths to these structures; particularly when we can see multiple initial stems fold to these structures. In addition in the revised manuscript we tried to better argue for these structures based on existing experimental evidence, such as the structural motifs found by Schlick et al. (denoted '3_6', coarse grained three stem structure invariant to stem length). We note that since the SHAPE datasets are created using bulk sequencing method, the SHAPE reactivity score may reflect the average reactivity of a large population of RNA molecules in different conformations, and may not reflect a specific structure.

7. Sanders et al. have a preprint (bioRxiv 2020 doi: 10.1101/2020.06.15.153197) on comparing structures in the same three viruses, including SHAPE-map data on all three. It's unclear why this data was not included in the analysis, it would appear to be a useful addition (especially the SHAPE data for MERS-CoV, which the authors did not analyze in the same way as SARS-CoV-2 because of the lack of SHAPE data).

   Response: Thank you for providing this reference. The data is not yet publicly available, we reached out to the authors to obtain it but have not yet heard back. Here we add to our discussion based on what we could learn from the existing information in the article: (1) "SHAPE data supports the existence of a pseudoknot at the same position in all three coronaviruses [72]" and (2) "This may be related to the overall trend that SARS-CoV and SARS-CoV-2 exhibited greater stable RNA structuredness across their genomes compared to MERS-CoV [72]."

8. The bracket notation for some of the structures appears to be wrong (some square brackets should be round brackets), making it impossible to tell in some cases which parts are pseudoknotted (the

pseudoknotted pairs are generally the only ones that are shown by square brackets, but many hairpins are shown with square brackets here). The "native" structure in Figs. 7 and 8 is just one example. The authors should carefully check their dot-bracket notation for all structures.

Response: As per the reviewer's suggestion we adjusted the dot bracket notation in the main text tables. We note that all added base pairs (regardless of being pseudoknotted or not) are presented in HFold and Iterative HFold as square brackets to show the last set of added base pairs. This was inherited in Shapify as well.

Minor points:

9. Overall, the manuscript is somewhat confusing because there are many different analyses being applied, and it's not always clear what result comes from what method. Clearer explanations would be helpful. Along the same idea, a schematic outlining the Shapify method (what each step is, which steps and/or datasets are used as inputs to which other steps, ...) would be an excellent idea for helping the reader understand.

   Response: Thank you for the feedback. In our revised manuscript we improved the structure of the paper to improve the clarity. We have added flowchart of Shapify as Figure S11 (supplemental file 1).

10. The motivation in the introduction for exploring alternative structures in pseudoknots (in terms of potential biological relevance) was clear and much appreciated. The authors might consider pointing out in the introduction that the structures that have been solved for the SARS-CoV-2 pseudoknot all have significant differences. This issue is discussed at the end, but it might be useful to highlight it in the introduction.

    Response: Thank you for the suggestion. We have added the following text to author summary: "Previous structural predictions obtained via different methods all have significant differences."

11. The "introduction" and "background" sections are overly long and contain repetitive sections. It is recommended that the authors combine them and make them more succinct.

    Response: As per reviewer's suggestion we revised the new Introduction section to include the information from the previous two sections and omitted unnecessary details.

12. On p. 5, additional evidence of similarities between different coronavirus pseudoknots, similar to Ref. 51, is seen in compounds that can inhibit frameshifting in several bat coronaviruses as well as human ones (also published in Viruses: Munshi et al. (2022) 14:177).

    Response: Thank you for providing this reference. We added the following text and citation to the Introduction: "Additional experiments find certain small molecules to be effective at inhibiting frameshifting for multiple coronaviruses–in both human and bat–showcasing the potential for pan-coronaviral therapeutic treatment design [41]."

13. One question about the importance of the initial stem: while clearly the thermodynamics of these stems are important, could the kinetics also not be important, given that the pseudoknots are unfolded as ribosomes pass over them and must refold in behind the ribosome? The kinetics of refolding after exiting the ribosome will complicate the analysis because they will disfavor the formation of hairpins involving pairing with the 3' end of the RNA sequence (which exits the ribosome last) and favor those that involve pairing exclusively in the 5' end (which exits first). The kinetics on the ribosome suggest that structures that require formation of hairpins in the 3' end are much less likely to be observed than the free energy would otherwise imply.

    Response: We added the following text in our Discussion section: "In addition it should be noted that kinetics may also play a role in initial stem formation. Specifically, refolding of RNA after the

ribosome passes over and unfolds the pseudoknot may favor pairing towards the 5' end which exits the ribosome first."

14. The language about secondary structure is somewhat confusing through the manuscript. Pseudoknots are usually considered a form of tertiary interaction; in addition to the pseudoknotted base-pairing, they often include triples, stacking, etc... One assumes that what the authors mean by secondary structure is the base-pairing interactions (all of them, including the pseudoknotted pairs); it would be helpful to clarify this point through a more explicit description.

    Response: We have clarified this point in the revised manuscript under "Background on RNA Structure Prediction" of the Materials and methods. Particularly we added the following text to better clarify the scope and definition of secondary structure: "We note that, while pseudoknotted base pairs are sometimes considered as part of the tertiary structure, we consider them as part of the secondary structure."

15. The SARS-CoV pseudoknots studied here dimerize via interactions in loop 2. Is dimerization considered in the analysis? It would presumably change the SHAPE values for the loop bases in the dimerization domain.

    Response: We thank the reviewer for their thoughtful comment and have added the following to discussion: "Here we acknowledge that tightness of the pseudoknotted loops caused by dimerization with other loops of the structure have been previously cited as possibly affecting correctness of SHAPE data [23]. However, in the SHAPE datasets we analyzed we could not find a non-reactive region (in two or more SHAPE datasets) that covers a loop region of the SARS-CoV-2 -1 PRF native structure."
