# Supplementary material for: Shapify: Paths to SARS-CoV-2 frameshifting pseudoknot
Source: PLoS Comput Biol. 2023 Feb 28;19(2):e1010922. doi: 10.1371/journal.pcbi.1010922 (PMC10004594; doi:10.1371/journal.pcbi.1010922)
Supplement: Attachment — Submitted filename: Response_to_Reviewers_Feb_2023.pdf [file pcbi.1010922.s003.pdf]

Response to Reviewers

February 1, 2023

We would like to thank the reviewers for their time and constructive feedback. We have revised the manuscript based on the feedback. Following is our point-by-point response to the comments with additions in blue.

Reviewer 1

The manuscript by Trinity et al., has been updated to reflect comments from other reviewers. It is now a well-expressed description of the Shapify algorithm used to predict RNA structures. The authors use existing RNA chemical reactivity (SHAPE) data and mutational analyses to demonstrate the strengths of their method over the existing Shapeknots method for analyzing the frameshift-stimulating pseudoknots of betacoronaviruses. RNA structures are not static, especially those processed by the ribosome, and the authors do a great job of presenting the possible structures elucidated in the pipeline they describe in the context of the known biology. There are two minor edits I would recommend prior to publication:

We thank the reviewer for the detailed feedback. Following we respond to each comment separately.

1. First, the phrase 'increased virility' in line 604 is not quite appropriate. I would recommend something like 'continued spread among various species'.

   Response: Thank you for the suggestion. We modified the text to "We hypothesize this may be a factor in the continued spread among various species of COVID-19."

2. Second, the authors mention kissing-hairpin structures in a couple of places (lines 506 and 578) that could be further investigated in the future. While these structures haven't been shown to facilitate frameshifting for the betacoronaviruses (SARS, MERS) analyzed in this work, similar structures that facilitate frameshifting been described for the alphacoronavirus 229E frameshift (Herold & Siddell, 1993, NAR 21:5838-42). It would not be out of place if the authors choose to add the analysis of elaborate internal structures present in larger frameshift-stimulating pseudoknots of the alphacoronaviruses to their future directions (line 588).

   Response: This is an interesting similarity with a group 1 coronavirus (SARS being group 2). Here we added the relevant citation and indication of future work. Modified to "These indicators point to possible kinetic paths for frameshifting pseudoknots that may be consistent across coronaviruses. Indeed, similar structures have been observed to initiate frameshifting in human coronavirus HCV 229E [73]. As a future direction we seek to further expand our structural analysis to additional coronaviruses and upstream targets including attenuator hairpin and palindromic sequences [74]."

Reviewer 2

In this work, Trinity et al. applied Iterative HFold to study SARS-CoV-2 -1 programmed ribosomal frameshifting (-1 PRF) structure, and adapted the program to develop Shapify by incorporating SHAPE reactivity data to guide hierarchical folding. To expand the knowledge of SARS-CoV-2 frameshifting pseudoknot, the

authors evaluated structural similarities between -1 PRF structures of SARS-CoV-2 and MERS-CoV, with a focus on predicted initial stems and suboptimal secondary structures. The authors also examined the effects of mutations on the -1 PRF structures from selected SARS-CoV-2 and MERS-CoV-2 mutated sequences, which exist in the population and have been validated experimentally for their effect on -1 PRF.The authors applied the Shapify pipeline to study the SARS-CoV-2 -1 PRF structure, obtained an ensemble of suboptimal structures and identified folding paths. For Shapify development, the parameters of the pseudo energy term from SHAPE reactivity data was determined from 30 RNAs with known structures and SHAPE datasets. The authors compared the performance of Shapify and the existing method ShapeKnots using four available SARS-CoV-2 SHAPE datasets (in vivo and in vitro). The authors revised the manuscript based on previous reviewers' feedback, including comparisons with existing method ShapeKnots and published results of -1 PRF structures. The Shapify pipeline is novel and unique in providing possible paths to the final folding. The scope of the study to identify notable non-native secondary structures and elucidate the structure-function relationship regarding -1 PRF is promising. However, these strengths could be better explained with improved figures and analyses. The manuscript seems to reach a publishable state.

We thank the reviewer for the detailed feedback. Following we respond to each comment separately.

1. The structural paths are presented in Figures 6 and 7 from initial stems to final foldings. The native pseudoknot 2D structure in Figure 6 seems to be a combination of initial stems 1, 2, and 3. It seems each of the three stems still exist in the final 2D structure that it reaches. Interestingly, initial stems 1 and 3 lead to the native or native-adjacent structure, but initial stem 2 reaches native non-adjacent structures. The finding might correspond to the -1 PRF mechanism, as it suggests possible folding pathways of the H-type pseudoknot. The authors mentioned related folding pathway study and initial stem 2's formation in Discussion. It might be helpful to elaborate the findings in this work and relate the paths in Figures 6 and 7 to biological functions.

   Response: Thank you for this insightful comment. We agree that this is an interesting finding. Here we added to the 'Incorporating SHAPE' discussion section "Interestingly, initial stem 2, which is energetically favourable, has multiple paths to different native non-adjacent structures. Further study is required to determine the effect of this mechanism in regulating frameshift efficiency."

2. Some initial stems in Table 4 might be located similarly and share common residues, for example, Stem 8 and 9. However, they might lead to different secondary structures in Figures 6 and 7. Does this suggest any base pair that might be critical to the stem in the final folding? It might be helpful to compare how base pairs in the initial stems are preserved in the final 2D structure.

   Response: With respect to Figures 6 and 7, it is difficult to determine if any specific base pair is critical in the final stem folding because the predictions are based on both initial stem constraints as well as SHAPE data. Furthermore, final predictions, especially those in Figure 7, present alternative final stem foldings and further complicate determining the effect of any single base pair. In order to allow easier cross-referencing of initial stems to final predictions, we have included two additional supplementary figures with initial stems highlighted in grey. Added: For additional visualization with initial stems highlighted, cf. S1 File, Fig. S12-13.

3. Using initial stems as constraints in ShapeKnots for paths might not be helpful, as the method is not the same with Shapify. The authors did mention this in their response to previous Reviewer 1 comment 3.

   Response: We included our previous response to reviewer in the Discussion subsection, Comparison with ShapeKnots. ShapeKnots predictions were not found to effectively utilize the initial stems (i.e., ShapeKnots performance was better without the initial stems as constraint).

Reviewer 3

I think the authors have addressed the scientific concerns that were raised in the previous rounds of review adequately.

Response: We thank the reviewer for the detailed feedback. Following we respond to each comment separately.

1. The clarity of the writing has been improved, but overall the manuscript still remains somewhat difficult to read. One thing that may help to ensure that the message of the paper is as clear as possible is to separate "discussion" from "conclusions", instead of combining them as done here. Please first discuss the implications of the results (along with any caveats and subtleties), and only after the discussion is finished present a brief (1-2 paragraphs) summary of the key results along with any concluding remarks in a separate "Conclusions" section.

   Response: Here we created a new Conclusions section, removing certain information from Discussion.

2. Line 5: Although ref. 3 reviews therapeutic approaches for Covid, it does not, in fact, discuss PRF as a therapeutic target, which is how it is used as a citation. The authors should cite a review that explicitly discusses targeting PRF therapeutically, some examples are Kelly et al. Virology (2021) (in the context specifically of coronaviruses) and Anokhina & Miller Acc Chem Res (2021) (in a broader context).

   Response: We have revised to instead cite Kelly et al. Virology (2021).

3. Line 303: under "Bootstrapping", I would recommend the authors avoid saying that they used a bootstrap "to generate additional information based on each original dataset", because bootstrapping cannot actually generate new information from a dataset!

   Response: We modified the sentence to avoid this misrepresentation. 'To further explore the degree to which the information in each SHAPE dataset was used in the structure prediction of Shapify and ShapeKnots, we implemented a bootstrap method as follows–for each original dataset, respectively.'
